# Motivating medical information system performance by system quality, service quality, and job satisfaction for evidence-based practice

**DOI:** 10.1186/1472-6947-12-135

**Published:** 2012-11-21

**Authors:** Ching-Sheng Chang, Su-Yueh Chen, Yi-Ting Lan

**Affiliations:** 1 R.O.C Naval academy, Kaohsiung City, Taiwan; 2 Department of Nursing, Kaohsiung Medical University Hospital, Kaohsiung Medical University, Kaohsiung City, Taiwan; 3 Postgraduate Programs in Management, I-Shou University, Kaohsiung City, Taiwan

**Keywords:** System quality, Service quality, Job satisfaction, System performance, AMOS 17.0 (structural equation modeling)

## Abstract

**Background:**

No previous studies have addressed the integrated relationships among system quality, service quality, job satisfaction, and system performance; this study attempts to bridge such a gap with evidence-based practice study.

**Methods:**

The convenience sampling method was applied to the information system users of three hospitals in southern Taiwan. A total of 500 copies of questionnaires were distributed, and 283 returned copies were valid, suggesting a valid response rate of 56.6%. SPSS 17.0 and AMOS 17.0 (structural equation modeling) statistical software packages were used for data analysis and processing.

**Results:**

The findings are as follows: System quality has a positive influence on service quality (γ_11_= 0.55), job satisfaction (γ_21_= 0.32), and system performance (γ_31_= 0.47). Service quality (β_31_= 0.38) and job satisfaction (β_32_= 0.46) will positively influence system performance.

**Conclusions:**

It is thus recommended that the information office of hospitals and developers take enhancement of service quality and user satisfaction into consideration in addition to placing b on system quality and information quality when designing, developing, or purchasing an information system, in order to improve benefits and gain more achievements generated by hospital information systems.

## Background

The rapid changes in the medical environment have greatly accelerated and increased hospitals’ demand for the quality and quantity of information processing. The increasing demand for information, tardy development of hospital information systems, and information personnel’s inability to keep abreast with technological advancements have jointly contributed to more neglected needs for information, outdated software crises and complaints among users [1,2]. The fact whether the information systems used by customer service personnel, administrative personnel, information personnel and medical personnel cater to their true needs affect the job satisfaction among these users and even their job performance is in dispute [1,3,4]. The issues on how to allow information systems to fulfill their management and policy-making support functions to meet the goals set by management and on how to decide the priority for the development of system functions with limited resources are what the medical policymakers would like to explore most [3-5]. Hence, understanding the effect of information system quality on service quality, job satisfaction and system performance and establishing indices for information systems will help hospital management make further decisions.

Since data of numerous types and huge quantities are generated everyday, accurately recording, rapidly delivering, and immediately processing a variety of tasks at hospitals for better medical services are the goals that each hospital strives for [2,3]. During the recent development of medical information systems, researchers targeting hospital information systems have gradually placed more b on the establishment of a complete hospital information system by achieving the balance between technology and humanity and serving different needs through effective communication [2,3,6]. Moreover, in the present day, where patient safety is concerned, the establishment, utilization and integration of hospital information systems (HIS) are an inevitable trend as well as an issue that modern medical institutions at different levels must face, in order to provide accurate clinical information for medical personnel, enhance the quality and efficiency of medical operations, ensure patients’ rights to seek medical care, and prevent medical malpractice [3,7,8]. These are also some of the focal points in developing hospital information systems.

In the field of information systems, many researchers have conducted relevant empirical studies with the DeLone and McLean’s [9] model of information systems success. However, DeLone and McLean [10] have reviewed and updated the model they previously proposed, adding a new construct, service quality, to the revised model, and combining individual impact and organizational impact into net benefits. As measurement is the foundation for management of information systems (MIS), in addition to investing a lot of resources in constructing a hospital information system, hospitals need an assessment model to evaluate and measure the quality and performance of the hospital information system constructed by hospitals [11]. From the perspective of system users, this study investigated the interactions between facilities and humanity in terms of four major constructs: system quality, service quality, job satisfaction and system performance. Moreover, this study aims to construct a medical information system assessment model that is suitable for the medical environment, in the hope of offering medical institutions a complete and objective tool and framework to evaluate medical information systems, helping medical institutions to better understand what system users truly need, improving the efficiency of clinical services, enhancing medical quality and safeguarding patient safety, while providing comprehensive evaluation and reference.

### Literature review

#### Relationship between system quality and service quality

In regard to the measurement items in the “service quality” construct, most studies have used the service quality (SERVQUAL) scale for measurement of service quality from the following aspects: tangibility, reliability, responsiveness, assurance and empathy. Li et al. [12] have suggested that factors such as information system personnel’s technical expertise and attitudes, product/service schedule, required time for system development, system alternation procedures, maintenance and support by suppliers, average processing amount of the information center, users’ knowledge of the system and educational training should be taken into consideration when evaluating service quality [13]. DeLone and McLean [9] have listed a total of 18 measurement items for evaluating system quality after reviewing 100 major research papers. In his study on the satisfaction among website users, McKinney et al. [14] have divided system quality into four aspects; the definition of each is as follows: 1. Accessibility: access to a website when connecting to the website at any time; 2. Usability: website layout design and the ease of use; 3. Navigation: availability of links to necessary information; and 4. Interactivity: personalized website design. These aspects all affect the service quality perceived by system users. In conclusion, the information department of an organization offers not merely products; services should be included as well. Thus, it is necessary to measure the effectiveness of the services provided by information systems [15]. Based on the discussions above, *Hypothesis 1* of this study is proposed: System quality has a positive influence on service quality.

#### Relationship between system quality and job satisfaction

Davis [16] has pointed out that in a technology acceptance model (TAM), the factors that affect system users are perceived usefulness and perceived ease of use of a system. Users’ perception affects their attitudes and even their behavioral intention and use behavior. According to the study by Holbrook [17], when a hospital system features security, ease of use and efficiency in terms of system quality, a positive attitude toward the system will be developed. Such an attitude will enhance job satisfaction, work performance and organizational commitment [18,19]. As far as job satisfaction is concerned, if high usefulness and ease of use are perceived by employees toward a hospital information system, the employees will have a positive opinion on the system, which will motivate them to utilize the system [20-22]. Due to its usefulness and ease of use, the system will benefit users to a certain extent at work, thereby enhancing their job satisfaction [23,24]. Similarly, the usefulness and ease of use of a system perceived by employees or users allow them to gain support and encouragement at work, thereby motivating them to devote more efforts to their job and even actively participate in work-related activities; consequently, employee’s devotion to their jobs is improved. DeLone and McLean [9] have reviewed and arranged the factors used to measure information systems success in the past, and proposed six constructs, including system quality, information quality and user satisfaction, believing that system quality and information quality both affect the satisfaction among information system users. Hence, *Hypothesis 2* is: system quality has a positive influence on job satisfaction.

#### Relationship between system quality and system performance

Two variables are included in the “system performance” construct: work performance and organizational commitment. Work performance refers to the yield and results generated by individual employees at work. The personal factors that affect work performance include: knowledge, skills, capabilities, motivation and attitudes. The transitional mechanism that helps yield better performance results at work includes: a performance management system, interactions with colleagues and superiors, definite performance goals, company encouragement, and reward measures or plans in recognition of outstanding performance [25]. Tax et al. [26] have defined performance as the speed by which an organization reaches its goal. Many studies have discovered that organizational commitment is a predictor to issues such as employees’ absence from duty or resignation, and there is a negative correlation between organizational commitment and these issues [27]. This discovery shows that employees who are highly committed to the organization and are willing to devote more efforts to achieving the organizational goals tend to remain within the organization and assist the organization in gaining a high reputation [28,29]. System quality is defined as the measurement of information systems, as it concerns programming errors in a system, user interface consistency, ease of use of user interface, documentation quality, as well as the quality and maintainability of software codes. DeLone and McLean [9] have believed that system quality and information quality simultaneously affect use, user satisfaction and individual performance and further influence organizational performance. In summary of the above, *Hypothesis 3* is: system quality has a positive influence on system performance.

#### Relationship between service quality and system performance

Rai et al. [30] have indicated that from the perspective of service quality, the goal of an organization is to provide customers with high quality services, and multiple processes are combined. The concept of service quality can be applied to information systems, since information systems can be regarded as a service function to deal with an organization’s need for information. The information department of an organization thus becomes a service provider. According to Parasuraman’s [31] definition, service quality is based on service users’ comparison between expected services and perceived services [32,33]. Zeithaml and Bitner [34] have suggested that the major decisive factors of expected services include: word-of-mouth communications, personal needs, past experience and communications between service providers and service users. Pitt et al. [35], based on the constructs developed by DeLone and McLean [9], argued that information systems success factors should include service quality; in other words, information quality, system quality and service quality influence system users’ satisfaction. Myers et al. [36] have proposed a complete framework to evaluate information systems success factors. Information quality, system quality, service quality and user satisfaction are among the eight constructs that they proposed. From the above studies, it can be learned that information quality, system quality, service quality and user satisfaction exert a significant influence on the evaluation of information systems success factors [37-39]. Based on the discussions above, this study assumes that a positive correlation exists between information system service quality and individual performance, and this correlation further affects organizational performance. Based on the discussions above, *Hypothesis 4* of this study is proposed: Service quality has a positive influence on system performance.

#### Relationship between job satisfaction and system performance

Researchers Biner et al. [40] have investigated the antecedents and consequences of job satisfaction among high-tech personnel. Their findings reveal that work performance is one of the consequences of job satisfaction; employees’ job satisfaction affects their work performance. Laudon and Laudon’s [41] study discussed the relationships of job satisfaction to performance, organizational commitment, relationship with manufacturers and intention to renew contracts. Their findings show that job satisfaction greatly influences the performance, organizational commitment, relationship with manufacturers, and intention to renew contracts. Pettit et al. [42] have studied the relationship between job satisfaction and work performance with a sample population consisting of 302 employees from two factories. The results showed that a significant positive correlation exists between job satisfaction and work performance. The early human relations school believed that high morale leads to high productivity [41,43]. This theory has given rise to many follow-up studies related to attitudes. Fishbein and Ajzen [44] have defined attitudes as the consistency in the preference and non-preference for certain things. Later, many studies on the relationship between attitude and behavior emerged, particularly on the relationship between job satisfaction (attitude) and work performance (behavior) [45]. Robbins [46] has also suggested that there is a direct relationship between job satisfaction and employees’ productivity [47]. Work performance may be improved by enhancing employee satisfaction. Hence, *Hypothesis 5* is: job satisfaction has a positive influence on system performance.

### Theoretical framework

**Figure 1:**
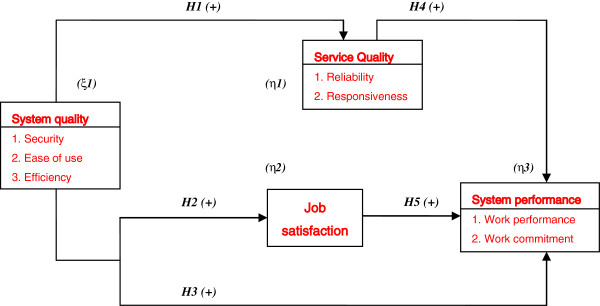
Conceptual framework of the relationship among system quality, service quality, job satisfaction, and system performance.

We thus derive a conclusion from the motive, purpose, and scholarly articles review that system quality has a positive influence on service quality (*Hypothesis 1*), job satisfaction (*Hypothesis 2*), and system performance (*Hypothesis 3*); service quality has a positive influence on system performance (*Hypothesis 4*); and job satisfaction has a positive influence on system performance (*Hypothesis 5*). Therefore, based on the literature review and hypotheses above, this study proposes an integrated research model (see Figure 1).

## Methods

### Research subject and data collection

We adopted a cross-sectional design using a questionnaire survey in this study. The study was approved by the managing supervisor of each surveyed hospital, and the convenience sampling method was applied to the information system users (employees) of three hospitals in southern Taiwan. The managing supervisor pasted a notice requesting volunteers for the anonymous questionnaire on bulletin boards at each surveyed hospital and made the questionnaires available for volunteer employees to fill out anonymously at information desk; respondent employees could ask questions directly to the trained personnel at information desk, and the personnel collected even those questionnaires not filled out at information desk and made reminders directly to those employees once a month. The response period was limited to two months. The authors invited all the volunteer employees to a seminar to explain the details of the study, and an introduction letter was attached to the questionnaire to explain the purpose of the study and to ensure respondent confidentiality. The questionnaire provided contact information so that respondents could later inquire about the results of the study. A total of five hundred questionnaires were distributed between May 2010 and July 2010, and three hundred and two were returned; 283 valid ones were collected after incomplete and incorrect questionnaires were filtered out, with a valid response rate of 56.6%. Israel [48] has proposed that the more samples drawn, the more statistical significance will be found (but probably the identification of this statistical significance will be meaningless from a management perspective, and it may lead to statistical misrepresentation), thus, they suggested that when the population size is more than 100,000, then, theoretically, the sample size should lie between 204 (95% confidence level and ± 7% precision) and 400 (95% confidence level and ± 5% precision). Therefore, the number of questionnaires gathered in this work is consistent with the theoretical sample size.

### Instrument development

A structured questionnaire was used as the measurement tool to collect research data in this study. First, empirical data from related literature and valuable opinions from medical informatics experts provided a basis for compilation of the draft questionnaire. Second, the draft questionnaire was revised and edited by three professors, two directors of the information office, two physicians, three nursing personnel and three general employees to create the initial questionnaire. Third, the initial questionnaire was reviewed by three professors and medical informatics experts who offered concrete suggestions in regard to the appropriateness and relevance of the questionnaire and revised the questionnaire again to ensure expert validity of the initial questionnaire. Finally, the questionnaire was then officially distributed after being completely compiled. This study adopted the questionnaire survey method, using a questionnaire as the major research tool. With the aim of helping participants to successfully complete the questionnaire, in terms of questionnaire design, a five-point Likert scale was used in the structured questionnaire except for personal information, and closed-ended questions were developed. The questionnaire is generally divided into two parts: (1) participants’ basic information; (2) four constructs. Because patients directly filled in the questionnaires in the independent variable and dependent variable sections, a single source bias (the deviation caused by the common method variance) might occur [49]. Thus, to avoid and reduce the occurrence of common method variance which might raise the possibility of overestimation and underestimation by the patients, we adopted: 1) a participant information confidentiality approach, using an anonymous method to reassure the participants; and 2) a concealed purpose approach, by not revealing the variables of every aspect in the questionnaire to reduce the doubts and suspicions that participants may have. Table 1 summarizes constructs and variables, including operational definitions for all variables. Questionnaires were examined for reliability and validity as follows:

1. Reliability analysis (exploratory factor analysis): Reliability refers to the accuracy and precision of measurement tools, including the stability and consistency of test results. As Table 1 illustrates, all Cronbach’s α values were 0.90 to 0.92, meeting the acceptable standard of more than 0.6, and no single factor included only one question [50,51].

2. Construct convergent validity (confirmatory factor analysis): The confirmatory factor analysis could gain higher recognition than expert content validity, and the results for all dimensions are listed in Table 2. All of the adequacy indicators were close to the ideal. Parameters (λ) between each latent variable and manifest variable were estimated to determine the significance of the estimated parameter (λ) in order to evaluate convergent validity. Thus, as Table 3 shows, the t values for the factor loading of all measurement items reached the level of significance (*p< .05*), and the composite reliability values for all constructs were greater than 0.6, which demonstrated satisfactory convergent validity [50,52,53].

**Table 1:** Summary of constructs and variables

**Construct**	**Variable**	**Operational definition**	**Cronbach’s α (> .6)**	**References**
**System quality**	Security	It refers to a hospital’s capabilities of providing information system services that safely protection the user information, confirmation the user identification, and prevention the virus requested by users.	0.90	[9,12]
	Ease of use	It refers to the degree to which the information system service is perceived as relatively easy and human-oriented to learn and use by users.		
	Efficiency	It refers to the degree to which the information system service is perceived as greatly helpful to facilitate and improve the work efficiency and work speed.		
**Service quality**	Reliability	It refers to a hospital’s capabilities of providing information system services that correctly delivery the service requested by users.	0.91	[31,38]
	Responsiveness	It refers to a hospital’s capabilities of providing information system services that instantly and rapidly respond to users’ demands.		
**Job satisfaction**	Job satisfaction	The psychological state of system user involves their positive or negative feelings or attitudes after having experienced information system services.	0.92	[40,47]
**System performance**	Work performance	It refers to the benefits such as strengthening users’ work efficiency, professional skills, operation process, etc. that information system services could provide.	0.91	[36,37]
	Work commitment	It refers to users’ inclination to remain within the hospital due to the capabilities of overcoming any problem encountered that information system services could provide.		

**Table 2:** Results of convergent validity analysis

**Indicator**	**System quality**	**Service quality**	**Job satisfaction**	**System performance**	**References**
*χ*2/df. (< 3)	2.82	2.72	2.70	2.88	[52]
GFI (> .9)	0.91	0.92	0.94	0.91	
AGFI (> .8)	0.88	0.90	0.92	0.87	
NFI (> .9)	0.90	0.92	0.95	0.90	[54]
RMSR (< .08)	0.063	0.070	0.068	0.079	[55]

*χ*^*2*^*/df.*, Ratio of Chi-square; *GFI*, Goodness of Fit Index; *AGFI*, Adjusted GFI; *NFI*, Normal Fit Index; *RMSR*, Root Mean Square of Standardized Residual.

**Table 3:** Results of confirmatory factor analysis

**Construct**	**Variable/Question item**	**Standard loading (*p< 0.05*)**	**Composite reliability (> 0.6)**	**AVE (> 0.5)**
**System quality**	***Security***		0.83	0.55
	1. User login is required to access the hospital information system.	0.73*		
	2. Auto logout is enabled after a period of inactivity on the hospital information system.	0.72*		
	3. The antivirus software prevents the hospital information system from being attacked by virus.	0.73*		
	4. The hospital information system is regularly maintained and examined by personnel from the Information Office.	0.78*		
	***Ease of use***		0.93	0.76
	1. The user interface of the hospital information system is easy to use.	0.84*		
	2. The tutorials or instructions provided by the hospital help me learn how to use the system easily.	0.98*		
	3. The user interface designed by the hospital is human-oriented.	0.93*		
	4. I am clear about every function of the hospital information system.	0.71*		
	***Efficiency***		0.94	0.78
	1. I am very familiar with the interface of the hospital information system.	0.86*		
	2. The user interface items of the hospital information system are quite easy to understand.	0.78*		
	3. The hospital information system greatly helps to improve work efficiency.	0.95*		
	4. The processing speed of the hospital information system affects my work speed.	0.94*		
**Service quality**	***Reliability***		0.82	0.53
	1. The data I entered is easily uploaded to the central processing system.	0.70*		
	2. The data from the hospital information system is trustworthy.	0.72*		
	3. The hospital information system is an essential tool that supports my work.	0.78*		
	4. The hospital information system can satisfy my individual needs.	0.71*		
	***Responsiveness***		0.95	0.81
	1. The hospital information system gives rapid warnings of error values entered.	0.94*		
	2. The hospital system detects problems rapidly and offers immediate support.	0.95*		
	3. The hospital information system provides tutorials or instructions.	0.84*		
	4. The hospital information system offers troubleshooting tips.	0.87*		
**Job satisfaction**			0.95	0.79
	1. The hospital information system improves my work performance. **(JS1)**	0.88*		
	2. The hospital information system enhances harmony between me and my supervisors (subordinates). **(JS2)**	0.96*		
	3. The hospital information system enhances the teamwork between me and my colleagues. **(JS3)**	0.85*		
	4. The hospital information system offers better achievements in work planning. **(JS4)**	0.89*		
	5. The hospital information system reduces my workload. **(JS5)**			
	6. I feel accomplished with the hospital information system. **(JS6)**	0.86*		
**System performance**	***Work performance***		0.95	0.79
	1. The information system strengthens my professional skills.	0.89*		
	2. I am capable of handling the mistakes made by the information system.	0.87*		
	3. I am familiar with the operation process of the information system.	0.86*		
	4. The information system improves my work efficiency.	0.95*		
	5. The information system allows me to achieve better results in the evaluation by the hospital.	0.88*		
	***Work commitment***		0.91	0.66
	1. I do my best to overcome any problem encountered when using the information system.	0.79*		
	2. Because of the complete functions of the information system, I will never consider leaving the hospital even when there is a better offer.	0.82*		
	3. I am confident of the hospital’s future due to the complete functions of the information system.	0.76*		
	4. Because of the complete functions of the information system, I am inclined to remain within the hospital even if the environment is getting worse.	0.83*		
	5. The environment provided by the information system and complete system motivate me to share my experiences with my colleagues.	0.85*		

3. Construct discriminant validity: This study performed discriminant validity analysis based on the recommendations of Bagozzi and Yi [52] and Hair et al. [50] by limiting the correlation coefficient of the paired dimensions to 1, then performing a Chi-square variance test of the limited and unlimited measurement patterns. If the Chi-square value of the limited pattern exceeds the Chi-square value of the unlimited measurement pattern and reaches a level of significance, then both dimensions have discriminant validity. Thus, as Table 4 shows, the Chi-square values of limited patterns in fact exceeded those of unlimited patterns, and reached a level of significance, indicating discriminant validities among all dimensions.

**Table 4:** Results of discriminant validity analysis

**PATTERN**	***χ***^***2***^	***d. f.***	Δ***χ***^***2***^
**System quality**			
Unlimited Measurement Pattern	55.21	62	-----
Security and Ease of use	143.26	63	88.05**
Security and Efficiency	131.57	63	76.36**
Ease of use and Efficiency	117.55	63	62.34**
**Service Quality**			
Unlimited Measurement Pattern	45.79	22	-----
Reliability and Responsiveness	131.22	23	85.43**
**Job Satisfaction**			
Unlimited Measurement Pattern	28.94	15	-----
JS1 and JS2	51.22	16	22.28**
JS1 and JS3	44.35	16	15.41**
JS1 and JS4	42.63	16	13.69**
JS1 and JS5	41.25	16	12.31**
JS1 and JS6	43.96	16	15.02**
JS2 and JS3	53.26	16	24.32**
JS2 and JS4	51.27	16	22.33**
JS2 and JS5	52.69	16	23.75**
JS2 and JS6	55.47	16	26.53**
JS3 and JS4	43.55	16	14.61**
JS3 and JS5	44.78	16	15.84**
JS3 and JS6	40.28	16	11.34**
JS4 and JS5	41.37	16	12.43**
JS4 and JS6	40.01	16	11.07**
JS5 and JS6	42.60	16	13.66**
**System performance**			
Unlimited Measurement Pattern	73.48	46	-----
Work performance and Work commitment	116.38	47	42.90**

** *p*< .01.

### Data analysis methods

The SPSS 17.0 and AMOS 17.0 (structural equation modeling) statistical software packages were used for data analysis and processing, including:

1. Descriptive statistical analysis: To determine the sample characteristics.

2. Structural equation modeling (SEM): According to Chang and Chang [56] and Joreskog and Sorbom [57], structural equation modeling clarifies the extent of relationships between variables as well as the chain of cause and effect. Restated, SEM results do not merely show empirical relationships between variables when defining the practical situation. For this reason, SEM was used to test the Hypotheses. This study also used several indices, including Chi-square ratio (< 3), goodness of fit index (GFI> .9), adjusted goodness of fit index (AGFI> .8), normal fit index (NFI> .9) and root mean square of standardized residual (RMSR< .08) to evaluate overall model fitness [58].

### Ethical considerations

Upon approval by the hospital Institutional Review Board, the study was then carried out with participants’ written consent; each participant’s personal data was kept anonymous and confidential and used only for research purposes (e.g. leaving out the participant phone number who had not completed the questionnaire at the information desk in order to make reminders directly to respondent employees for collection of the questionnaire and destroying the participant phone number after the questionnaire had been collected etc.) to comply with the spirit of the Declaration of Helsinki, 2008. The response period was limited to two months. An introductory letter was attached to the questionnaire to explain the purpose of the study and to ensure respondent confidentiality. Anyone who was also interested in learning about the results of this study was able to request a copy through the contact address provided in the questionnaire.

## Results

### Characteristics of samples

Table 5 shows the demographic data of the sample population in this study. In terms of gender distribution, the female population accounted for 84.5% of the total population. The ages of the participants mostly ranged between 21 and 30 years (48.8%), while 91.5% of hospital employees were below the age of 40. 77% of the personnel had a junior college degree or above. The questionnaires completed by physicians took up 9.2% of the total questionnaire copies. Response rate of nursing personnel was the highest in this study, accounting for 53%. The personnel other than medical personnel were regarded as administrative personnel (others) in this study, with a response rate of 33.2%. As for seniority distribution of the participants, most of them had 0–5 years of seniority (69.6%). The job descriptions of the participants (or the units they work for) were mostly related to administrative management, accounting for 32.9%, while most of the participants assumed non-director positions, with a ratio of 96.8% (see Table 5).

**Table 5:** **Characteristics of samples (*N*= 283)**

**Description**	**Frequency**	**Percentage (*%*)**
***Gender***		
Male	44	15.5
Female	239	84.5
***Age***		
20 years and below	3	1.0
21-30 years	138	48.8
31-40 years	118	41.7
41-50 years	17	6.0
51 years and above	7	2.5
***Education***		
High school and below	65	23.0
Junior college	71	25.0
Bachelor’s degree	123	43.5
Master’s/Doctorate degree	24	8.5
***Position***		
Director	9	3.2
Non-director	274	96.8
***Title***		
Physician	26	9.2
Pharmacist	13	4.6
Nurse	150	53.0
Others	94	33.2
***Seniority***		
0-5 years	197	69.6
6-10 years	55	19.4
11 years and above	31	11.0
***Job Description (Unit)***		
Outpatient management	53	18.7
Hospitalization management	33	11.7
Medical records management	42	14.8
Examination and blood bank management	20	7.1
Medicine management	16	5.7
Administrative management	93	32.9
Others	26	9.1

### Structural equation modeling (SEM)

As Table 6 illustrates, all the hypotheses in this study were also demonstrated to be statistically significant. System quality had a positive influence on service quality (γ_11_= 0.55, *hypothesis 1*), job satisfaction (γ_21_= 0.32, *hypothesis 2*), and system performance (γ_31_= 0.47, *hypothesis 3*). Service quality (β_31_= 0.38, *hypothesis 4*) and job satisfaction (β_32_= 0.46, *hypothesis 5*) positively influenced system performance. Table 6 shows the results of SEM in this study and the model goodness of fit. In short, it can be concluded that the research model is applicable for the data.

**Table 6:** Results of structural equation modeling

**Path**	**Path name**	**Path coefficient**	***t *Value**
System quality (ξ1)→ Service quality (η1) **(*H1*)**	γ11	0.55	6.40*
System quality (ξ1)→ Job satisfaction (η2) **(*H2*)**	γ21	0.32	3.11*
System quality (ξ1)→ System performance (η3) **(*H3*)**	γ31	0.47	5.56*
Service quality (η1)→ System performance (η3) **(*H4*)**	β31	0.38	3.65*
Job satisfaction (η2)→ System performance (η3) **(*H5*)**	β32	0.46	5.26*
System quality (ξ1)→ Security (x1)	λ1	0.053	1.07
System quality (ξ1)→ Ease of use (*x*2)	λ2	0.65	7.83*
System quality (ξ1)→ Efficiency (x3)	λ3	0.41	4.33*
Service Quality (η1)→ Reliability (y1)	λ4	0.031	0.79
Service Quality (η1)→ Responsiveness (y 2)	λ5	0.60	7.22*
System performance (η3)→ Work performance (y 3)	λ6	0.59	7.03*
System performance (η3)→ Work commitment (y 4)	λ7	0.46	5.22*
Goodness of fit *χ*^2^/*d.f.*= 2.39, GFI = .92, AGFI = .87, NFI = .92, RMSR = .056.			

* *p* < .05.

## Discussion and conclusions

While computer technology is extensively applied to handle affairs, hospital information system managers are facing a critical issue as to how to establish an information system that is suitable for hospitals to obtain optimal efficiency and benefits. Evaluation of information systems offers an important approach to determine whether a unit using the systems enables the existing facilities to function to the fullest extent possible. During the process from determining research objectives, developing a research framework and performing empirical analyses to obtaining the research findings, a better understanding has been achieved pertaining to a variety of hypotheses previously developed, and several conclusions and discussions are made, which are provided separately below:

1. Relationship among system quality, service quality, job satisfaction, and system performance

Our findings support the statement that system quality will positively influence service quality. This agrees with the assertions of previous relevant studies. For example, Keating et al. and McKinney et al. [14,15] have pointed out that system quality could affect the service quality perceived by system users. Our findings also support the statement that system quality has a positive influence on job satisfaction. This agrees with the assertions of previous relevant studies. For example, Davis and DeLone and McLean [9,16] have pointed out that system quality and information quality both affect the satisfaction among information system users. Other cases in which some past researchers’ viewpoints corresponded to the results of this study that system quality has a positive influence on system performance, for example; Asghari and Aissa and Babulak [25,28] have showed that system quality and information quality simultaneously will affect system performance. The results show that only security, one of the system quality factors, has an insignificant influence on service quality, job satisfaction, and system performance. A possible reason is that most of the system users are physicians, nursing personnel and pharmacists who are less familiar with the safety measures that protect the system, unlike professional information personnel. Consequently, no significant influence of security can be detected on service quality, job satisfaction, and system performance in the analysis of the questionnaires.

This study also found that ease of use and efficiency about perceived system quality had significant influence on service quality, job satisfaction, and system performance. Information technology helps medical institutions to offer rapid, efficient and accurate medical services; however, information system users’ lack of professional knowledge regarding medical information technology as a result of their non-information related background, the innovative computer and information technology and increasing dependence on information have increased the workloads of information personnel. Under such circumstances, ease of use and efficiency become more important for enhancing information system service quality.

2. Relationship between service quality and system performance

Our findings support the statement that service quality will positively influence system performance. This agrees with the assertions of previous relevant studies. For example, Myers et al. and Pitt et al. [35,36] have pointed out that service quality could affect the organizational system performance. The research findings indicate a significant positive influence of service timing and personnel responsiveness on system performance. The results also show that reliability, one of the service quality factors, has an insignificant influence on system performance.

A possible reason is that hospital employees are unfamiliar with information systems, and one of the potential causes of the departments’ unfamiliarity with information systems is that the systems developed by contractors are unable to meet users’ needs; individual and system performance is thus affected. More and more attention has been paid to the effect of information systems on administrative management performance and clinical performance. However, information system’s effect on teaching performance and research performance has not been recognized by hospital system users. Therefore, this is probably because the participants possess more computer operating skills than the knowledge with regard to knowledge management activities, such as how information systems can be utilized to support teaching and research. The reasons behind the results are issues that require each hospital’s attention and contemplation, and it is recommended that academia and hospitals join forces in the future to carry out related research and improvements.

3. Relationship between job satisfaction and system performance

Our findings also support the statement that job satisfaction has a positive influence on system performance. This agrees with the assertions of previous relevant studies. For example, Laudon and Laudon and Pettit et al. [41,42] have pointed out that system performance may be improved by enhancing employee satisfaction. The results reveal that when employees are more satisfied with their jobs and love their jobs more, they devote more efforts to their jobs, leading to improved system performance. A significant positive causal relationship exists between these two factors.

Thus, information systems have a comprehensive influence on hospitals. These research findings may provide a basis for future hospitals to develop a new information system or improve the existing system, and may serve as reference for the information industry in developing high quality hospital information systems to enhance management efficiency and effectiveness. The research findings also offer a measurement tool to investigate whether the units using information systems allow the existing facilities to function to the fullest extent possible and to determine the benefits generated by hospital information systems. Consequently, medical care services that feature high quality, high efficiency and reduced medical care costs may be provided through analyses and improvement plans.

### Suggestions

As verified by the empirical results, the success of hospital information systems does not merely rely on hardware equipment or software programs; administrative support in every aspect is also an important factor. It is necessary to acquire computer-related knowledge for information system users. Only when users are familiar with the process, functions and objectives of hospital information systems and adapt to changes in work processes or methods brought by information systems can information systems optimize their efficiency at work, information technology provide full support, operational procedures be accelerated, workloads be reduced, work quality be improved, and more achievements be gained with hospital information systems. As a result, in addition to the possession of expertise, personnel in charge of hospital systems should comply with the user orientation principles, which mean to respect users’ needs and opinions, in order to create an information system that meets individual needs and organizational performance goals. It is thus recommended that the information office of hospitals and developers take enhancement of service quality and user satisfaction into consideration in addition to placing b on system quality and information quality when designing, developing, or purchasing an information system, in order to improve the benefits generated by hospital information systems.

### Managerial implications

Employees believe that when they provide better quality of services, their work performance is improved as well. Hospital information systems offer intangible services. The design must be based on the perspective of system users in order to develop high quality system services that meet users’ needs and allow users easier access. By so doing, improved employee and organizational performance will surely become the subsequent results of enhanced service quality. One of the critical factors that lead to success or failure of an information system is the way the system is developed and maintained. Due to the differences in the internal scale and actual demands of each hospital, the ways in which information systems are developed and maintained are also different. With limited manpower and financial resources, regional and local hospitals (small and medium hospitals) still tend to outsource the hospital information system development project or purchase package software. Nonetheless, there is a quality gap among system developers or contractors. Under such circumstances, hospitals ought to be more cautious when choosing a contractor, and pay more attention to its techniques, capabilities, after-sales services, experiences and understanding of hospital demand. Moreover, hospitals need to provide a clear description of the specifications required, while contractors have to come up with a set of reasonable plans or theories regarding the operating process or method for the developed hospital information system, in order to maximize outsourcing benefits, effectively reduce management costs, enhance information users’ satisfaction, and increase overall net benefits.

### Research limitations and future studies

Despite all the efforts that have been committed to a strict process in the construction of the research framework and selection of the research methods, this study is subject to certain environmental factors and subsequently some limitations, as follows:

1. This study examined a non-random convenience sample of information system users in a single country, and should be generalized cautiously to other populations. However, given the context of the study, the survey results exhibited adequate validity and reliability.

2. The questionnaire can merely probe into the attitudes participants hold toward the questions. To some degree, while the questions may lead to some subjective answers, it is difficult to derive participants’ true opinions on the subject. Thus, we suggest future researchers conduct in-depth interviews with the participants and utilize quantitative and qualitative approaches to obtain a more definitive result.

3. Cooper and Schindler [59] and Culyer and Newhouse [60] have proposed that using aggregation data for inference of individual behaviors might lead to biases. When individual data cannot be observed, using the average value for inference would easily result in biases, because average values cannot reflect individual differences. Therefore, the demographic variables (characteristics of the respondents) were taken as the control variables in this study, but it is expected that individual medical difference can be used as the unit of analysis in future studies to estimate its flexible influence, and hence to conclude the differences in various aspects (constructs) between different hospitals or different specialist departments, etc.

4. Finally, this study examined only one period, which would not reveal factors with long-term effects. A multiple period approach is suggested for follow-up study. Analyzing multiple periods of data would achieve more complete and objective statistical data.

## Competing interests

The authors declare that they have no competing interests.

## Authors’ contributions

First author CS led the development of this manuscript and contributed to the research design, methodology, and revised draft. Author SY provided the important opinions for the revised draft and secured grant funding. Author YT provided the important opinions for the revised draft and collected the questionnaires. All authors approved and read the final draft.

## Pre-publication history

The pre-publication history for this paper can be accessed here:

http://www.biomedcentral.com/1472-6947/12/135/prepub
